# Exploring the Association Between Cervical Microbiota and HR-HPV Infection Based on 16S rRNA Gene and Metagenomic Sequencing

**DOI:** 10.3389/fcimb.2022.922554

**Published:** 2022-06-21

**Authors:** Bingyan Fang, Qun Li, Zixian Wan, Zhenbo OuYang, Qiushi Zhang

**Affiliations:** ^1^ The Second School of Clinical Medicine, Southern Medical University, Guangzhou, China; ^2^ Department of Gynecology, Guangdong Second Provincial General Hospital, Guangzhou, China; ^3^ Graduate Collaborative Training Base of Guangdong Second Provincial General Hospital, Hengyang Medical School, University of South China, Hengyang, China

**Keywords:** 16S rRNA gene sequencing, metagenomic sequencing, HR-HPV, cervical microbiota, cancer

## Abstract

The relationship between the cervico-vaginal microbiome and high-risk human papillomavirus (HR-HPV) is well observed. However, there is a lack of adequate research regarding the cervical microbiota in HR-HPV infection. Most published research results have used 16S rRNA gene sequencing technology; this technology only focuses on marker sequences, resulting in incomplete gene information acquisition. Metagenomic sequencing technology can effectively compensate for the deficiency of 16S rRNA gene sequencing, thus improving the analysis of microbiota function. Cervical swab samples from 20 females with HR-HPV infection and 20 uninfected (Control) women were analyzed through 16S rRNA gene and metagenomic sequencing. Our results indicated that the composition and function of the cervical microbiota of HR-HPV infection differed notably from that of control women. Compared with control women, Firmicutes was decreased during HR-HPV infection, whereas Actinobacteria was increased. At the genus level, Lactobacillus was enriched in control women, while levels of Gardnerella and Bifidobacterium were lower. At the species level, *Lactobacillus crispatus*, *L. jensenii*, and *L. helveticus* were enriched in control women; these were the top three species with biomarker significance between the two groups. Eight pathways and four KEGG orthologies of the cervical microbiota of statistical differences were identified between the HR-HPV infection and control women. Collectively, our study described the cervical microbiota and its potential function during HR-HPV infection. Biomarkers of cervical microbiota and the changed bacterial metabolic pathways and metabolites can help clarify the pathogenic mechanism of HR-HPV infection, making them promising targets for clinical treatment and intervention for HR-HPV infection and cervical carcinoma.

## Introduction

Cervical carcinoma is the world’s fourth most common cancer in women, the incidence of which is rising and the trend is getting younger (Ma et al., 2020). Prevention and therapy of cervical lesions is currently an urgent problem requiring solution (Theresa, 2017). Research indicates that persistent HR-HPV infection is a major risk element for cervical precancerous lesions and cervical cancer (Mitra et al., 2016). When infected with HPV, the biological shield created by immune microecology of the reproductive tract is destroyed, leading to the proliferation of abnormal microbiota (Anderson, 2007). Imbalance of the microflora of the reproductive tract, on the one hand, will inactivate tumor suppressor genes p53 and Rb by increasing the expression of HPV oncogenes E6 and E7, and then promote the progression of the cervical epithelium to malignant lesions (Anderson, 2007). On the other hand, it will increase the adherence, intrusion, and colonization of aberrant microbiota, which will eventually lead to the aggravation of imbalance, forming a vicious cycle and ultimately promoting the occurrence of cervical cancer (Kwasniewski et al., 2018; Bi et al., 2020). At present, the cause of persistent HR-HPV infection is not clear, and some studies suggest that personal discrepancies in immune function or genetic susceptibility are at work, but the determining element is possibly cervico-vaginal microecology (Mitra et al., 2016; Kyrgiou et al., 2017; Chao et al., 2019a).

The microenvironment of the female reproductive tract has a dynamic balance composed of a large number of microbiotas with self-regulation (Curty et al., 2020). Among them, the dominant Lactobacillus can resist pathogenic infections by producing lactic acid, bacteriocin, and biosurfactants (Witkin and Linhares, 2017). Microbiota of the female reproductive tract will change under the influence of physiological or external factors, and when it exceeds the range of physiological regulation, the microecological balance is destroyed (Aniewski et al., 2019). HR-HPV infection has been found to be associated with dysbacteriosis in the reproductive tract (Aniewski et al., 2019). HR-HPV infection also leads to reproductive tract microbiota disorder; at the same time, microbiota imbalance makes females more susceptible to HR-HPV infection (Martinez et al., 2021), nevertheless the pathogenesis still requires further clarification.

Previous studies regarding microbiota are mostly based on traditional methods of microbial culture; however, most microbiota cannot be isolated and cultured in a laboratory, resulting in limited experimental data, and a prolonged heavy workload (Arokiyaraj et al., 2018; Chao et al., 2019b; Wei et al., 2020). Since the concept of “Metagenome” was first proposed by Handelman in 1998, sequencing technology based on metagenome has developed rapidly (Tringe and Rubin, 2005), and research related to microbiota has been further deepened from phenotypic and genotypic identification (Zou et al., 2020). At present, metagenomic research is mainly achieved by 16S rRNA gene and metagenomic sequencing. The former is currently the most widely used technology in the study of microbiota, with the advantages of no culture, simplicity, speed, and low cost (Ranjan et al., 2016). As 16S rRNA gene sequencing is aimed at the sequencing of marker genes, complete genomic information of bacteria cannot be obtained; the resolution of bacterial identification level is usually accurate only at the genus level, and detailed analysis of bacterial functions cannot be carried out (Marchesi and Ravel, 2015). Metagenomic sequencing is sequencing analysis of the total DNA of a sample, the depth of species identification can reach the species level, and the function and metabolic pathways of microbiota can be further studied (Shah et al., 2018). This technology makes up for deficiencies in 16S rRNA gene sequencing in the depth, accuracy, and functional analysis of species identification. As most previous studies have been based on 16S rRNA gene sequencing, only a few studies have used metagenomic sequencing for analysis, which describes the association between cervico-vaginal microbiota and HR-HPV infection, therefore more comprehensive and accurate data is needed.

The core of research regarding reproductive tract microecology is microbiota. Most of the previous studies focused on vaginal microbiota. Studies indicate that the diversity of the cervical microbiome is higher than that of the vagina, and changes of the cervical microbiome in patients with HR-HPV infection are more obvious than those in the vagina (Zhai et al., 2021). Therefore, this study combined 16S rRNA gene and metagenomic sequencing analyze the composition and function of cervical microbiota between HR-HPV infection and control women, then further explored the relationship between cervical microbiota and HR-HPV infection, in the hope of revealing the mechanism of HR-HPV infection and cervical lesions. The aim was to inhibit the development of persistent HR-HPV infection and cervical lesions by rebuilding the microbial balance of the reproductive tract.

## Method

### Subject Selection

We recruited as participants, 40 women of reproductive-age who visited the Department of Gynecology or Physical Examination Center at Guangdong Second Provincial General Hospital and who met the inclusion criteria, from December 2020 to September 2021. Based on the results of HPV DNA, cervical thinprep cytologic tests, and cervical biopsy pathology, the participants were divided into two groups: 20 women infected with HR-HPV (HR-HPV) and 20 uninfected women (Control; all the participants cervical cytology thinprep cytologic test or cervical biopsy pathology showed no cervical intraepithelial lesions or malignant cells). All subjects were aged 25–45 y, had engaged in normal sex for one year or more without using hormonal contraception, and were not in their menstrual period, pregnant, or entering menopause. Exclusion criteria were (1) use of antibiotics or vaginal antimicrobials in the past month, and vaginal intercourse or vaginal lavage within the last 3 d; (2) suffering from bacterial vaginosis, candida vaginitis, urinary tract infectious diseases; (3) history of mycoplasma, chlamydia, gonorrhea, syphilis, genital herpes, trichomonas, or other sexually transmitted diseases; (4) previous treatment history of cervical lesions or total hysterectomy; (5) history of systemic diseases such as diabetes, autoimmune disease, and malignant tumors; (6) habits of smoking or drinking; (7) history of HPV vaccination. All subjects volunteered to participate in our research and provided written informed consent. The research was permitted by the ethics committee of the Second Peoples Hospital of Guangdong Province, and the research was carried out based on the principles of the Declaration of Helsinki.

### Specimen Collection and DNA Extraction

Three sterile swab specimens were taken from the cervical canal during a gynecological examination, and stored immediately at −80 °C for DNA extraction. Total genome DNA from specimens was extracted using the Hexadecyltrimethy Ammonium Bromide (CTAB) method. DNA concentration and purity was monitored on 1% agarose gels. Some DNA was destined for 16S rRNA gene sequencing, and the remainder for metagenomic sequencing.

### 16S rRNA Gene Sequencing and Bioinformatics Analysis

The V3–V4 region of the bacteria 16S ribosomal RNA gene were amplified by PCR (95 °C for 1 min, followed by 30 cycles at 98 °C for 10 s, 50 °C for 30 s, and 72 °C for 30 s, and a final extension at 72 °C for 5 min) using primers 341F 5′- CCTAYGGGRBGCASCAG-3′ and 806R 5′-GGACTACNNGGGTATCTAAT-3′. Sequencing libraries were generated using TruSeq^®^ DNA PCR-Free Sample Preparation Kit (Illumina, USA) following the manufacturer’s recommendations, and index codes were added. Finally, the qualifying libraries were sequenced on an Illumina Novaseq 6000 platform to obtain 250 bp paired-end reads.

The raw data was quality-filtered and chimera sequences were removed to obtain high-quality clean data by QIIME (Backulich et al., 2013). Using the Uparse algorithm, effective sequences were clustered into different Operational Taxonomic Units (OTU) with 97% consistency by default (UPARSE, 2013). The OTU sequences were carried out using a Silva Database and the Mothur algorithm to annotate microbiota (Wang et al., 2007). Analysis of Alpha and Beta diversities were performed using QIIME (v.1.7.0) and QIIME (v.1.9.1), respectively.

### Metagenomic Sequencing and Bioinformatics Analysis

Library preparation for metagenomic sequencing was performed using the NEBNext^®^ Ultra DNA Library Prep Kit for Illumina (NEB, USA). The quality of the library was assessed by Agilent 2100. All specimen structured libraries were sequenced on an Illumina Novaseq 6000 platform (Illumina). The sequencing strategy was PE150, and the amount of sequencing data for each sample was 6G.

The raw sequences were cleaned by clearing the sequences which contained low quality bases, and the reads with unknown bases “N”. Then, the sequences were aligned to the human genome database to filter out the reads that may originate from the host using Bowtie v.2.2.4 software (Karisson et al., 2013). Subsequent data was selected for further analysis. Kraken2 software, that used K-mers precise alignment and streamlined database methods, was used to annotate species based on reads mapping (Wood and Salzberg, 2014). HUMAnN2 software was used for functional and microbial pathway abundance profiling of metagenomic samples based on the Kyoto encyclopedia of genes and genomes (KEGG) Database (Abubucker et al., 2012).

### Statistics

The SPSS v.25.0 software was used to analyze the participants’ general data. Wilcoxon rank-sum test was used for intergroup comparison of the 16SrRNA gene and metagenomic sequencing. Statistical analysis was performed with R software (v.2.15.3). *P* < 0.05 was considered to indicate a statistically significant difference.

## Result

### General Data of Subjects

The research included 20 females infected with HR-HPV (“HR-HPV” group) and 20 healthy females (“Control” group). There was no obvious difference in age (*P* = 0.318), BMI (*P* = 0.811), age at first sexual encounter (*P* = 0.058), or Parity (*P* = 0.236) between the two groups (**Table 1**).

**Table 1:** Characteristics of subjects in the control and HR-HPV groups.

Characteristics	Control (n=20)	HR-HPV (n=20)	*P* ^a^ *-*value
Age (y) ^b^	35.55 ± 4.12	33.75 ± 6.78	0.318
BMI (kg/m^2^) ^b^	21.92 ± 3.12	21.68 ± 3.19	0.811
Age at first sex (y) ^b^	22.80 ± 1.64	21.65 ± 2.06	0.058
Parity ^c^	18/20(90%)	14/20(70%)	0.236

The P ^a^-value were obtained by Chi-square analysis and Students t-test. ^b^Mean ± SD ^c^ n/N(%) BMI: body mass index

### 16S rRNA Gene Sequencing Characteristics

**Figure 1:**
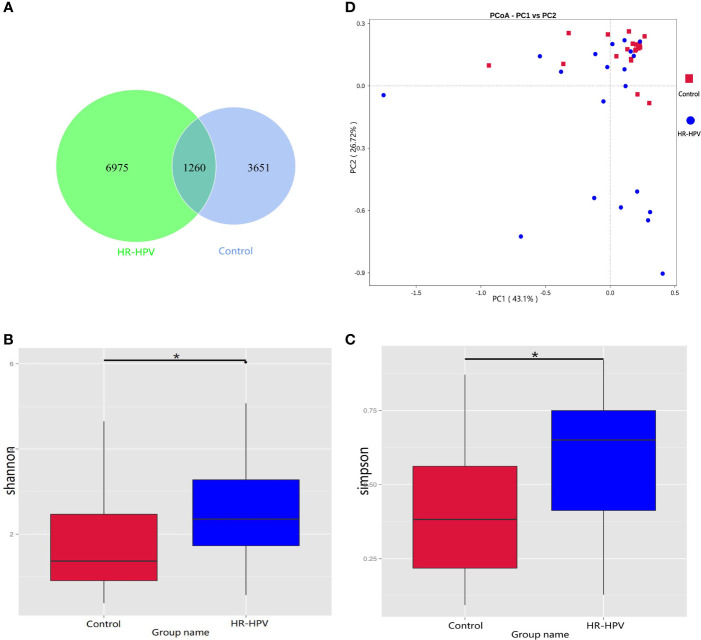
16S rRNA gene sequencing characteristics. **(A)** Venn graph of cervical microbiota between the two groups. **(B, C)** Comparison of the alpha diversity based on Shannon and Simpson indices between the two groups (*P < 0.05). **(D)** PCoA based on Weighted Unifrac distance.

We obtained 3 350 704 raw reads from 40 subjects’ cervical swab samples. After removing barcodes, primers, low-quality bases, short sequences, and chimeras, 2 909 768 effective reads were obtained with an average efficiency rate of 86.63%. A total of 4911 OTUs in Control and 8235 OTUs in HR-HPV were achieved with 97% consistency (**Figure 1A**). Alpha diversity analysis suggested that the Shannon and Simpson index was significantly higher in HR-HPV than in Control (**Figures 1B, C**). Beta diversity was used to further understand the similarities and differences between samples. The principal coordinate analysis (PCoA) based on Weighted Unifrac distance showed that there was a trend of separation between samples in HR-HPV and Control. (**Figure 1D**). The Wilcoxon test suggested significant differences in the structure of cervical microbiota between the two groups (*P*<0.05).

### Microbial Composition Based on 16S rRNA Gene Sequencing

**Figure 2:**
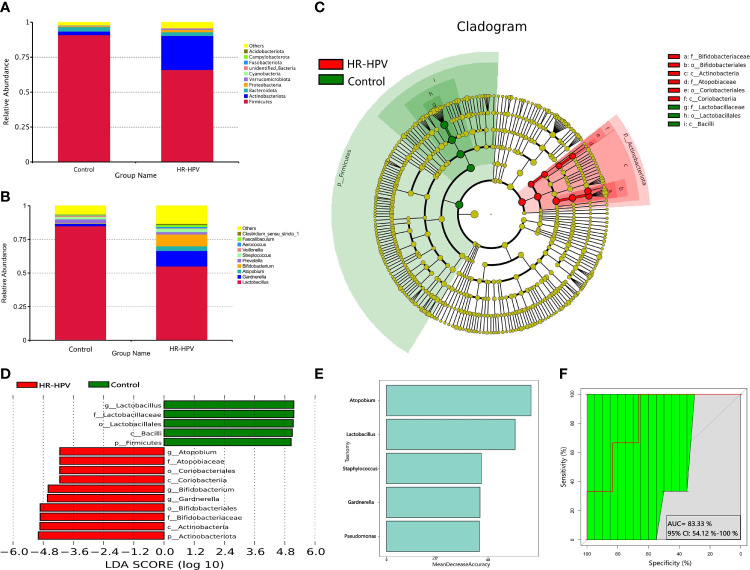
Microbial composition based on 16S rRNA gene sequencing. **(A)** Relative abundance of the cervical microbiota at the phylum level. **(B)** Relative abundance of the cervical microbiota at the genus level. **(C)** LEfSe cladogram by 16S rRNA gene sequencing of the cervical microbiota in the two groups. Microbial composition was compared at different species levels, from the phylum in the outermost ring to family in the innermost ring. **(D)** Distribution histogram of LDA score assessed for species with biomarker significance between the HR-HPV infected and uninfected women (*P*<0.05; LDA score >4). **(E)** The importance of species at the genus level in the predictive random forest model using the mean decreasing accuracy. **(F)** ROC curve generated by random forest model predicting five genera in the cervical microbiota. The plots shown in the ROC represent the corresponding optimal threshold.

Firmicutes was the most dominant phylum in the two groups, followed by Actinobacteriota. Compared with Control, the relative abundance of Firmicutes in HR-HPV was obviously decreased, while Actinobacteriota had the opposite results (**Figure 2A**). At the genus level, Lactobacillus was most dominant in Control, accounting for 84.90%, followed by Gardnerella (1.76%), Atopobium (0.21%), and Bifidobacterium (0.34%). In HR-HPV, the relative abundance of Lactobacillus decreased significantly, accounting for only 54.98%, while the relative abundance of Gardnerella (11.59%), Atopobium (3.43%), and Bifidobacterium (8.76%) increased (**Figure 2B**).

Linear discriminant analysis (LDA) effect size (LEfSe) analysis was performed to identify the biomarkers of cervical microbiota between HR-HPV and Control. At the phylum level, Firmicutes was higher in Control, while Actinobacteriota was enriched significantly in HR-HPV. At the genus level, the cervical microbiome of HR-HPV was highly enriched with Gardnerella, Bidfidobacterium, and Atopobium, while Control was enriched with Lactobacillus (LDA score >4; **Figures 2C, D**).

Five trials of tenfold cross-validation of the random forest classifier models between Control and HR-HPV were constructed to identify diagnostic biomarkers. Within every cross-validation in each trial, 70% of the data was used as a training set while the remaining 30% was used as a test set to evaluate performance. The operating characteristic curve was used to evaluate the greatest discriminant model, and five biomarkers were found (**Figures 2E, F**).

### Metagenomic Sequencing Characteristics

We obtained 1 693 961 998 raw reads from metagenomic sequencing, with a total of around 254.09 G data, with an average of 6.35G per sample. Approximately 253.66 G of clean data were obtained by fine-filtering low-quality bases and undetected bases in raw data. A total of 1.48 G data were obtained through host contamination filtering of clean data, with an average of 0.37 G for each sample. The ratio of the data before and after quality control was 0.58%, indicating that the samples were highly contaminated by the host, resulting in a small amount of data filtered by the host and quality control. If the analysis strategy that an assembly-based approach is used, poor or even difficult gene assembly may occur, leading to errors in data interpretation. Therefore, Kraken2 and HUMAnN2 softwares, based on a read-based (mapping) approach, were selected for analyzing microbial composition and function, respectively, which solving the problem of less effective data owing to serious host contamination.

### Microbial Composition Based on Metagenomic Sequencing

**Figure 3:**
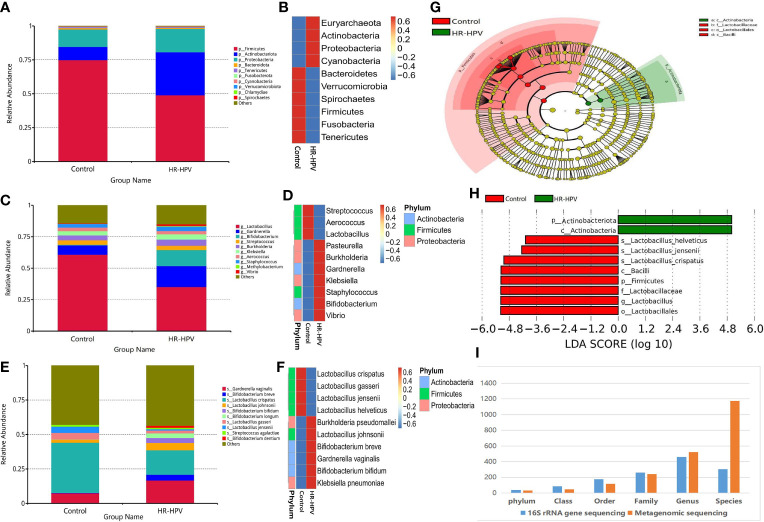
Microbial composition based on metagenomic sequencing. **(A)** Relative abundance of the cervical microbiota at the phylum level. **(B)** Cluster heatmap of relative abundance at the phylum level. **(C)** Relative abundance of the cervical microbiota at the genus level. **(D)** Cluster heatmap of relative abundance at the genus level. **(E)** Relative abundance of the cervical microbiota at the species level. **(F)** Cluster heatmap of relative abundance at the species level. **(G)** LEfSe cladogram by metagenomic sequencing of the cervical microbiota in the two groups. Microbial compositions were compared at different species levels, from phylum in the outermost ring to family in the innermost ring. **(H)** Distribution histogram of LDA score assessed for species with biomarker significance between the HR-HPV infection and uninfected women (*P*<0.05; LDA score >4). **(I)** Comparison of the accuracy of identifying bacteria between the 16S rRNA gene and metagenomic sequencing.

Effective sequences were annotated by Kraken2 metagenomic classification software, to further explore the cervical microbial composition of the two groups. The relative abundance of Firmicutes was decreased in HR-HPV, while Actinobacteria was increased (**Figures 3A, B**). Compared with Control, the relative abundance of Lactobacillus decreased, and Gardnerella, Bifidobacterium were increased in HR-HPV (**Figures 3C, D**). These results were in agreement with previous 16S rRNA gene sequencing. At the species level, *L. crispatus* was the dominant species in Control, accounting for 36.39%. The relative abundance of *L. crispatus* (17.74%) decreased significantly, while *Gardnerella vaginalis* increased in HR-HPV (**Figures 3E, F**). Alpha diversity, according to the Shannon and Simpson indices, did not differ between the two groups; the alpha diversity of cervical microbiota in HR-HPV did not differ from Control.

LEfSe analysis revealed that differential microbiota at the phylum level were consistent with 16S rRNA gene sequencing. Lactobacillus was greatly enriched at the genus level in Control. At the species level, *L. crispatus, L. jensenii*, and *L. helveticus* were the top three biomarkers with significant differences in Control (LDA score > 4; **Figures 3G, H**).

### Comparison of the Accuracy of Identifying Bacteria

The comparison of the number of species annotated by 16S rRNA gene and metagenomic sequencing at different taxonomic levels showed that there was no significant difference at the phylum, class, order, family, and genus level (**Figure 3I**). At the species level, there were 1174 species annotated by metagenomic sequencing, while only 304 by 16S rRNA gene sequencing; the former was obviously higher than the latter. These results verified that the accuracy of identifying bacteria only reach the genus level using 16S rRNA gene sequencing, while it can reach the species level when using metagenomic sequencing.

### Metabolic Functions of Cervical Microbiota

**Figure 4:**
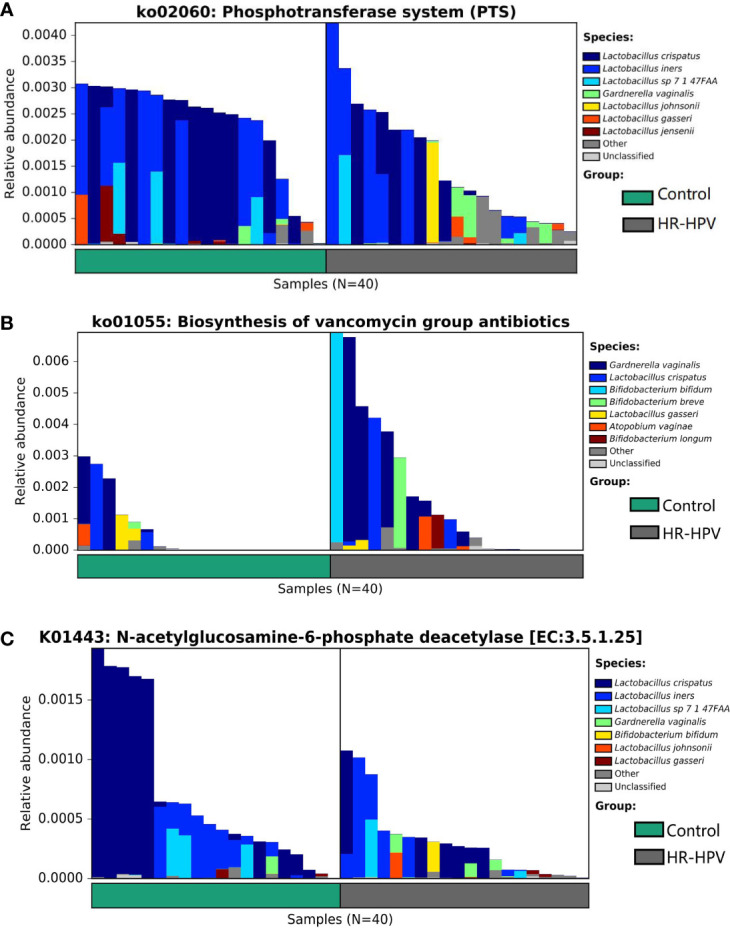
The analysis of functional annotation of the KEGG database combined with the relative abundance of cervical microbiota. **(A)** Analysis of the species contribution degree of ko02060 **(B)** Analysis of the species contribution degree of ko01055 **(C)** Analysis of the species contribution degree of K01443.

The analysis of microbial function and metabolism was based on the KEGG database using HUMAnN2 software. There were 240 KEGG pathways detected, 30 KEGG pathways were obviously different in abundance between groups, including eight pathways with relative abundance >0.01%. Among them, the relative abundance of ko03010 was dominant, but there was no obvious difference. ko00052, ko00520, ko00627, ko02060, ko03320, ko04930, and ko05340 were enriched in Control, while ko01055 was enriched in HR-HPV (**Table 2**). ko02060 and ko01055, which were significantly enriched and had the highest abundance in Control and HR-HPV groups, respectively, were selected for species contribution analysis. The results showed that the enrichment of ko02060 decreased significantly with the decreased abundance of *L. crispatus* and *L. iners*, the increase of *G. vaginalis* and *L. johnsonii* in HR-HPV (**Figure 4A**). With the increase of the abundance of *G. vaginalis* and Bifidobacterium, the enrichment of ko01055 rose obviously in HR-HPV (**Figure 4B**).

**Table 2:** Relative abundance of nine KEGG pathways and four KEGG orthologies (KOs).

KEGG pathway	Pathway Name	Control	HR-HPV	*P*-value	KEGG Orthology	KO Name	Control	HR-HPV	*P*-value
ko00052	Galactose metabolism	0.0018	0.0013	0.0167					
ko00520	Amino sugar and nucleotide sugar metabolism	0.0021	0.0016	0.0460	K02777	PTS system, sugar-specific IIA component [EC:2.7.1.-]	0.0002	0.0002	0.0409
					K01443	N-acetylglucosamine-6-phosphate deacetylase [EC:3.5.1.25]	0.0007	0.0003	0.0227
ko00627	Aminobenzoate degradation	0.0002	0.0001	0.0402					
ko01055	Biosynthesis of vancomycin group antibiotics	0.0005	0.0018	0.0184					
ko02060	Phosphotransferase system (PTS)	0.0023	0.0016	0.0181	K02777	PTS system, sugar-specific IIA component [EC:2.7.1.-]	0.0002	0.0002	0.0409
ko03320	PPAR signaling pathway	0.0002	0.0001	0.0181					
ko04930	Type II diabetes mellitus	0.0002	0.0001	0.0499					
ko05340	Primary immunodeficiency	0.0004	0.0003	0.0375					
ko03010	Ribosome	0.0106	0.0078	0.0859	K02970	small subunit ribosomal protein S21	0.0004	0.0003	0.0326
					K02913	large subunit ribosomal protein L33	0.0200	0.0130	0.0583

Of the 3673 KEGG orthologies (KOs) detected, 234 were found to be statistically significant. K01443 with the highest relative abundance was selected for species contribution analysis. With the decreased abundance of *L. crispatus* in HR-HPV, the enrichment of K01443 also decreased significantly (**Figure 4C**). Of the 234 KOs, four KOs were involved in these KEGG pathways (**Table 2**). K01443 and K02777, which were involved in ko00520 were decreased in HR-HPV, as were K02970 and K02913, which were involved in ko03010, and K02777, which was involved in ko02060. K02913 had the highest abundance in the two groups, mainly enriched in the HR-HPV group, without obvious difference.

## Discussion

In our research, we compared the composition and function of the cervical microbiome in HR-HPV infection and uninfected subjects using 16S rRNA gene and metagenomic sequencing. There were obvious differences in distribution and function of microbiota between the groups, including three species and eight pathways. Further, four KOs within those pathways were identified.

The results of cervical microbiota composition at the phylum and genus levels in 16S rRNA gene sequencing were basically consistent with metagenomic sequencing, which further verified the precision of 16S rRNA gene sequencing. At the species level, the abundance of *L. crispatus* decreased in the HR-HPV group, whereas *G. vaginalis* was increased, even similar to *L. crispatus*. LEfSe analysis was used to further explore the diagnostic biomarkers associated with HR-HPV. The prediction of biomarkers by the random forest model further validated the results of LEfSe at the genus level, and also highlighted the importance of Staphylococcus and Pseudomonas. *L. crispatus*, *L. jensenii*, and *L. helveticus* were the prominent three species with biomarker significance. Previous cross-sectional and longitudinal research indicates that the vaginal microbiome predominated by *L. crispatus* and is more common in women without HPV infection or cervical lesions (Marconi et al., 2020). At the same time, many studies have found that vaginal microbiomes predominated by *L. crispatus* have a positive impact on infertility, bacterial vaginosis (BV), and other gynecological diseases (France et al., 2016; Gottschick et al., 2017; Chen et al., 2021). Another study has shown that a supernatant cultured with *L. crispatus* and *L. rhamnosus* can reduce the expression of autophagy genes ATG14, BECN1, and HPV E6 oncogene to create an anti-proliferative effect on HeLa cervical cancer cells (Motevaseli et al., 2016). The study also found that the supernatants of *L. gasseri*, *L. crispatus*, and *L. jensenii* can downregulate the expression of HPV oncogene E6 and E7, cyclin A, and cyclin-dependent kinase 2 (CDK2) to inhibit the activity of cervical cancer cells (Ghanavati et al., 2020). *L. helveticus* is widely used in various types of food processing, and relevant studies based on *L. helveticus* and HR-HPV infection have not been reported. In conclusion, we hypothesized that the reduction of dominant species in cervical microbiota of females infected with HR-HPV weakens the inhibition of pathogenic bacteria, allowing them to proliferate and subsequently promote the occurrence and progression of disease. *L. crispatus* and *L. jensenii* may be protective factors of HR-HPV infection and cervical lesions.

The results of 16S rRNA gene sequencing indicated that the richness and diversity of cervical microbiota in females infected with HR-HPV were significantly increased, in agreement with previous research (Eun et al., 2013; Nieves-Ramirez et al., 2021). However, another study showed that the cervical bacterial richness of HPV-positive females was less than for HPV-negative females (Wu et al., 2020). According to our metagenomic sequencing, the alpha diversity between the two groups showed no obvious difference. The inconsistencies of the two sequencing results may be owing to different sequencing methods (Eun et al., 2013; Shah et al., 2015).

Based on the KEGG database, HUMAnN2 analysis indicated that among the metabolic pathways significantly enriched in Control, the phosphotransferase system (PTS) is the main mechanism for bacteria to absorb carbohydrates. The abundance of PTS was positively associated with that of *L. crispatus* and *L. iners*, but negatively correlated with that of *G. vaginalis*. The PPAR signaling pathway involves nuclear hormone receptors activated by fatty acids and their derivatives; a study suggested that genetic variations in this pathway gene may contribute to pancreatic cancer susceptibility (Liu et al., 2020). It has been found that PDIA3 siRNA can suppress proliferation, invasion, and migration of AML cells through amino sugar and nucleotide sugar metabolism (Ye et al., 2018). A study indicated that the biosynthetic pathway of peptidoglycan is enriched in cervical cancer patients (Kwon et al., 2019), but this pathway was without obvious difference among groups in our study. We determined that four KOs that were statistically significant between groups were involved in these pathways, including N-acetylglucosamine-6-phosphate deacetylase, sugar-specific IIA component, small subunit ribosomal protein S21, and large subunit ribosomal protein L33, which were enriched in the Control women. These discoveries only illustrated that the function and metabolites of cervical microbiota in HR-HPV infection differed obviously from that in Control women; the effect of this on HR-HPV infection remains to be further explored through experiments and combined metabolic analysis.

Following the in-depth research on reproductive tract microbiota in recent years, it is now known that dysbacteriosis contributes to the occurrence and progress of HPV infection, cervical lesions, and even cervical cancer. Therefore, the intervention and treatment of reproductive tract microbiota disorder has been a breakthrough for preventing cervical cancer and removing HPV. Its main purpose is to remove the pathogenic bacteria, and rebuild the balance of reproductive tract microecology to prevent further development of the disease and promote its cure. In an uncontrolled study of 35 HPV-positive women, oral administration of the probiotic *L. crispatus* M247 significantly altered vaginal microbiota types and increased HPV conversion rates in women (Nardini et al., 2016). In a prospective study of HPV infection, a lactobacillus vaginal application (*L. rhamnosus* BMX 54) group of 6-months had a much higher HPV conversion rate than a 3-month group, with a statistically significant difference (Recine et al., 2016). The use of probiotics can rebuild the microbial community structure of the reproductive tract dominated by Lactobacillus, improve the cure rate of bacterial vaginosis and other diseases caused by dysbacteriosis, and reduce their recurrence rate (Kovachev et al., 2014). With the continuous advancement of the “Human microbiota project”, the research and development of microecological drugs has become a research hotspot of domestic and foreign pharmaceutical enterprises (Dreher-Lesnick et al., 2017). SMMM (Small Molecule Microbiota Modulator) is the main research direction at present, which regulates microbial structure and metabolism or influences the mutual effect between the microbiome and host through precise intervention of the target identified in microbial research (Arnold et al., 2021; Abdel-Magid, 2019). Clarification of the relationship between reproductive tract microbiota and HR-HPV persistent infection, which can offer a scientific theoretical basis for the use of probiotics and microecological drugs, which is expected to be applied in clinical practice.

There were some limitations to this study. Differentiation studies between persistent HR-HPV infection and transient infection were ignored. Subsequently, the illustration of a causal relationship between HPV infection and microbiota by cross-sectional studies alone is insufficient; cohort studies should be performed to identify the potential impact of cervical microbiota biomarkers and altered metabolic pathways.

In conclusion, our study is the first in this field to report that combined 16S rRNA gene and metagenomic sequencing can more comprehensively demonstrate the diversity, microbial composition, and function of cervical microbiota between HR-HPV infected and uninfected women. Analysis using metagenomic sequencing can compensate for the lack of 16S rRNA gene sequencing technology. Meanwhile, the results of metagenomic and 16S rRNA gene sequencing gave mutual verification. Our study confirmed that the composition and function of the cervical microbiome in HR-HPV infection differed notably from that of uninfected women. *L. crispatus*, *L. jensenii*, and *L. helveticus* are the predominant three species with biomarker significance between the groups, and they represent potential microbial targets for future treatment. There were some metabolic pathways and metabolites of cervical microbiota that varied significantly; whether these affect the development of HR-HPV infection in women remains unclear. With the rapid development of molecular biology in recent years, the integration of multi-omics techniques such as metagenomics and metabolomics is promising. We plan to further clarify the causal relationship between HPV infection and the cervical microbiota and metabolites through combined metabolomics techniques in the near future.

## Data Availability Statement

16S rRNA sequencing data for all the samples have been deposited in NCBI with the accession number of PRJNA846153. Metagenomic sequencing data for all the samples have been deposited in NCBI with the accession number of PRJNA847258.

## Ethics Statement

The studies involving human participants were reviewed and approved by The ethics committee of Guangdong Second Provincial General Hospital. The patients/participants provided their written informed consent to participate in this study.

## Author Contributions

QZ and BF conceived the study question, and all authors were involved in the study design. BF and QL selected subjects and collected all the samples. BF, QL, ZW and ZO performed the sequencing and data analysis. BF created the first draft of the manuscript. QZ provided guidance throughout the study. All authors contributed to the article and approved the submitted version.

## Conflict of Interest

The authors declare that the research was conducted in the absence of any commercial or financial relationships that could be construed as a potential conflict of interest.

## Publisher’s Note

All claims expressed in this article are solely those of the authors and do not necessarily represent those of their affiliated organizations, or those of the publisher, the editors and the reviewers. Any product that may be evaluated in this article, or claim that may be made by its manufacturer, is not guaranteed or endorsed by the publisher.
